# HPV Detection in Head and Neck Squamous Cell Carcinomas: What Is the Issue?

**DOI:** 10.3389/fonc.2020.01751

**Published:** 2020-09-15

**Authors:** Jeremy Gbenakpon Augustin, Charles Lepine, Aurelien Morini, Anais Brunet, David Veyer, Camille Brochard, Haitham Mirghani, Hélène Péré, Cécile Badoual

**Affiliations:** ^1^Department of Pathology, Henri Mondor Hospital, APHP, Créteil, France; ^2^Department of Pathology, European Georges Pompidou Hospital, APHP, Université de Paris, Paris, France; ^3^INSERM U970, Université de Paris, Paris, France; ^4^Equipe Labellisée Ligue Contre le Cancer, Paris, France; ^5^Department of Virology, European Georges Pompidou Hospital, APHP, Université de Paris, Paris, France; ^6^Department of Head and Neck Surgery, European Georges Pompidou Hospital, APHP, Université de Paris, Paris, France

**Keywords:** HPV, DNA hybridization, RNA hybridization, p16, RNAscope, PCR, head and neck, squamous cell carcinoma

## Abstract

Besides classic tobacco and alcohol risk factors, human papillomavirus (HPV) plays a role in the development of a subset of head and neck squamous cell carcinomas (HNSCCs), and notably oropharynx squamous cell carcinomas (OPSCCs). HPV-induced OPSCCs have a different biological behavior and a better prognosis compared to non-HPV-induced OPSCCs and the eighth-edition TNM classification now separates these two entities. Therefore, determining the HPV status of patients with OPSCC is now essential for treatment, prognosis, and development of clinical trials. In this review, after reminding essential steps of HPV implication in the cell cycle, we describe the existing tools that are currently feasible in routine practice according to facilities available in health structures, with their benefits and drawbacks: HPV PCR, E6/E7 mRNA RT-PCR, E6/E7 mRNA *in situ* hybridization, HPV DNA *in situ* hybridization, and P16 immunochemistry. Besides these traditional HPV detection tools, novel diagnostic approaches are being evaluated for HPV-induced OPSCC “ultrastaging.” E6 humoral response and ddPCR-detecting HPVct DNA are two techniques performed on blood and are therefore non-invasive. Baseline E6 humoral levels could have a prognostic value, and HPVct DNA could be helpful for HPV OPSCC recurrence monitoring. At last, next-generation sequencing (NGS)-based “capture HPV” is a technique feasible on biopsies and circulating DNA material. It helps characterize HPV integration status and sites, and it could define prognostic subgroups in HPV-induced OPSCC. These novel precision detection tools could be further integrated in the care of patients with HPV-induced OPSCC.

## Introduction

Head and neck squamous cell carcinomas (HNSCCs) constitute a group of malignant tumors located in the oropharynx, larynx, hypopharynx, nasopharynx, and oral cavity. All together, they represent approximately 800,000 new cases and 400,000 deaths per year (1). Classic risk factors include tobacco and alcohol exposure, but it is now established that human papillomavirus (HPV) plays a major role in the development of oropharyngeal squamous cell carcinomas (OPSCCs) (2–5). This role is not so clear in non-oropharyngeal squamous cell carcinomas (non-OPSCCs), but some reports suggest a possible association between HPV infection and nasopharyngeal carcinomas (6, 7). HPV infection is found in 20–60% of the OPSCCs depending on the countries (8) with, for example, approximately 20% of HPV-induced OPSCCs in Bangladesh and South China (9, 10) and higher rates of HPV-induced OPSCCs in Western Europe and North America (11). Subdividing the HNSCCs in oropharyngeal and non-oropharyngeal carcinomas is therefore well integrated now, because of their different carcinogenesis. HPV-induced OPSCCs tend to occur more often in non-smokers and are associated with more frequent nodal involvement (4, 5). Previous studies reporting the HPV-induced OPSCC occurrence mostly in younger patients seem now to be countered by recent reports revealing that they can also develop at a later age under certain geographic and sociosexual conditions (12–14). Moreover, HPV-induced OPSCCs have a better prognosis than non-HPV-induced OPSCCs, with a better sensitivity to radiations and a better overall survival (5, 15). More broadly, HPV-induced OPSCCs have a better prognosis regardless of the modality of treatment (16–18). For HPV-positive non-OPSCCs, some subgroups might also have a better prognosis, but studies are heterogeneous and controversial (19). Because of these significant biological and clinical differences, HPV-induced OPSCCs have their own classification in the eighth edition of the UICC TNM classification (Union for International Cancer Control) (20). In this context, determining HPV status in HNSCCs and especially in OPSCCs has become mandatory. Besides, several trials based on radiation de-escalation programs or on immunotherapy are evaluating performances of treatments according to HPV status in OPSCCs, and it is essential to adequately classify patients (16, 21–23). Interestingly, the College of American Pathologists has recently published guidelines for HPV testing in HNSCCs (24). These recommendations focus on diagnostic tests in routine practice, and many of them are based on expert consensus opinion. According to these guidelines, all OPSCC samples should be tested for HPV. In this review, we will present the different tests currently used and give an insight into novel diagnostic approaches currently available in research but that could be further used in routine practice.

## HPV Involvement in the Cell Cycle

HPV involved in mucosal cancer can be divided into two main groups, depending on their oncogenic associated risk. Low-risk HPV are very rarely associated with the development of cancers, and HPV-induced OPSCCs are usually developed after a high-risk HPV infection. Conversely, high-risk HPV genotypes encompass HPV16, HPV18, HPV31, HPV33, HPV35, HPV39, HPV45, HPV51, HPV52, HPV56, HPV58, HPV59, and HPV68. The high-risk genotypes produce E6 and E7 oncoproteins. E6 protein binds to tumor suppressor p53 by the formation of a trimeric complex E6/E6AP/p53 (25), leading to the proteolytic degradation of p53 (26, 27). E7 protein binds to pRb (phosphorylated retinoblastoma protein), releasing E2F transcription factor and then promoting cell-cycle progression, and consecutively to p16 overexpression. Briefly, p16 is a CDK (cyclin-dependent kinase) inhibitor. This protein is involved in the pRB pathway, implicated in cell-cycle regulation. p16 protein has a cell-cycle regulation role by inhibiting the S phase. It is important to underline that interaction between p16 with CDK4/6 avoids CDK4/6-cyclin D complex formation and phosphorylation of Rb. Overall, p16 overexpression avoids phosphorylation of Rb family members, leading to capture of E2F by Rb proteins and thus to cell-cycle arrest into the G1 phase (28). Low-risk HPV produce E6 and E7 proteins which have lower affinity for p53 and pRb proteins (29) and thus are not theoretically associated with cell-cycle progression, nor with p16 overexpression. Nevertheless, no study has systematically studied the patterns of p16 expression in OPSSCs associated with low-risk HPVs. The reason why high-risk HPV-induced cancers overexpress p16 protein has been partially answered by studying epigenetic changes in HPV16 E7-expressing human epithelial cell lines (30). Independent of its function to inhibit pRB, E7 oncoprotein is responsible of KDM6B demethylase upregulation, leading to decreased levels of repressive H3K27me3 marks in the p16INK4a-encoding CDKN2A promoter region, responsible for the overexpression of the p16 protein. At last, maintenance of an HPV malignant phenotype (e.g., promotion of proliferation and prevention of apoptosis) in established HPV16-positive human OPSCC cell lines requires E6 and E7 proteins, as shown by Rampias et al. using shRNA targeting E6 and E7 transcripts (31).

## Main Techniques Used to Detect HPV in OPSCC

According to recent studies, based on this well-known molecular characteristic of the HPV virus to drive the cell toward a tumoral phenotype, different techniques have been developed. They tend to certify the HPV implication in OPSCC tissues: PCR (HPV DNA detection), RT-PCR (E6 and E7 mRNA detection), p16 immunohistochemistry, *in situ* hybridization targeting DNA (DNA ISH), and *in situ* hybridization targeting RNA (RNA ISH) (Table 1). All these assays have different advantages, diagnostic performances, and counterparts that we will detail further. These recommended routine diagnostic tests are completed to classify OPSCC HPV-positive (HPV+) or HPV-negative (HPV-) and other new performant biomarkers seem to be adapted for HPV-induced OPSCC ultrastaging. Indeed, as we already described before, the E6 and E7 HPV oncoproteins are responsible for cell transformation and carcinogenesis and have been proven to be indispensable for the maintenance of tumor phenotype (32). According to the CAP guidelines, every diagnosis of OPSCC should be followed by an assay evaluating HPV infection status in the tissue (24). Several techniques are available, depending on diagnostic performances and resources available in the laboratory. Optimal HPV detection should consider assays detecting (i) transcriptionally active infections, because transient infection does not seem sufficient to develop a carcinoma (33–35) and (ii) consistency with high-risk HPV, because those are associated with malignant processes (3, 4, 36). The 2017 revised WHO/IARC (World Health Organization/International Agency for Research on Cancer) recommendations introduced direct HPV testing based on *in situ* hybridization and/or PCR and/or anti-p16 immunochemistry to classify the OPSCC according to HPV status (37).

**TABLE 1:** Description of the benefits and drawbacks of different HPV detection techniques.

Detection technique	Benefits	Drawbacks	References
HPV PCR	High sensitivity HPV genotype information FFPE manageable Easy and inexpensive technique	No information about viral transcription High risk of contamination (intrinsic and extrinsic)	(49–60)
E6/E7 mRNA RT-PCR	High sensitivity and specificity Detects active viral infection Gold standard for research	Time-consuming Non-FFPE manageable (fresh or frozen tissue only) RNA fragility	(39–45)
E6/E7 mRNA *in situ* hybridization	High specificity and good sensitivity *In situ* detection of a transcriptionally active HPV infection FFPE manageable	RNA degradation over time Expensive technique	(62–65, 69–72)
HPV DNA *in situ* hybridization	*In situ* detection of HPV DNA High specificity FFPE manageable	Reduced sensitivity (needs a minimum DNA copy number)	(54, 62–67)
P16 immunochemistry	High sensitivity Inexpensive technique FFPE manageable	Moderate specificity Surrogate marker of HPV infection	(8, 62, 63, 70, 71, 81, 82) (87, 88, 92–95)
Serology for antibodies against E6 protein	Present in more than 90% of patient with OPSCC related to HPV16 Easy to set up	Lack of clinical data and hindsight	(119–124, 126)
HPV circulating tumoral DNA by ddPCR	Correlation with clinical outcome Early detection of recurrences in posttreatment monitoring High sensitivity and specificity Low cost	Need to be validated on larger cohorts	(52, 117, 130, 133)

*HPV, human papillomavirus; RT-PCR, reverse-transcriptase polymerase chain reaction; ddPCR, droplet digital PCR; FFPE, formalin-fixed paraffin-embedded tissues.*

### Molecular Assays

#### mRNAE6/E7 Detection

The maintenance of the transformed phenotype of HPV-driven tumor cells is based on the expression of E6 and E7 proteins (33–35). Therefore, detecting E6 and/or E7 protein expression constitutes the best tool to define a tumoral sample as an HPV-driven tumoral tissue or not. However, for the time being, performant techniques based on reliable immunohistochemical probes to detect such viral protein on tissue sample are not current. A recent study compared the results of E6 protein detection in lymph-node fine-needle aspirates, and oral samples (saliva or swabs) by OncoE6^TM^ Oral Test (Arbor Vita Corp©) to reference tests performed on FFPE material: p16 and high-risk HPV mRNA. Agreement between fine-needle aspirates OncoE6^TM^ and FFPE p16 was good (kappa = 0.53). Agreement between oral samples and FFPE p16 and high-risk HPV mRNA was poor (kappa = 0.02 for both), probably due to lower concentrations of E6 protein in these analytes (38). Thus, using such commercial assays on minimally invasive lymph-node fine-needle aspirates could be helpful to diagnose high-risk HPV infection in routine practice. Detection of E6 and/or E7 mRNA by RT-PCR on fresh/frozen samples is considered by some authors as the gold standard to diagnose an HPV-related OPSCC, particularly based on its capacity to represent an eventual prognosis biomarker (39). Nevertheless, it is important to be cautious about the accuracy and reliability of techniques detecting mRNA by RT PCR regarding available samples. Even if the accuracy of this technique has been tested on formalin-fixed paraffin-embedded (FFPE) samples (40), such assays should be used on fresh/frozen tissues given the better diagnostic performances obtained with these types of samples when compared to FFPE ones (41–44). This may be mostly explained by higher RNA destruction and fragmentation of FFPE samples and subsequent decreased sensibility of RNA detection by RT-PCR techniques. Therefore, the gold standard E6/E7 mRNA detection for HPV-related OPSCC diagnosis requires fresh samples (45) and is not useful for routine screening as it is technically demanding. However, a recent study about the development and the validation of a novel and rapid molecular detection method for HR-HPV in FFPE tumor tissues based on combined HPV DNA and E6 mRNA detection reached an accuracy of 97 and 100%, respectively, in OPSCC and oral cavity squamous cell carcinoma (46).

#### PCR and HPV Genotyping

Firstly and until now, several commercially available assays have been clinically validated on cervical swabs to detect high-grade preneoplastic lesions (47, 48). However, none of these commercial molecular assays have been specifically validated for clinical routine practice on OPSCC samples. Most of these assays target the L1 gene and amplify a region from 65 to >400 bp according to the technique. Different studies in small cohorts of patients have demonstrated the possibility of using these assays for HPV detection in OPSCC on fresh tissues. These techniques are known to be stable and reproducible, and a recent meta-analysis found the pooled sensitivity and specificity of HPV DNA PCR to be respectively 98 and 84% for HPV detection in OPSCCs (49). However, FFPE samples are often the only material available for molecular testing after pathological examination in the OPSCC context and only few studies have evaluated different commercial molecular assays on head and neck FFPE biopsies (50–55). Regarding the frequent proportion of degraded DNA in FFPE samples, some authors such as Steinau et al. suggest to pretreat FFPE tissues using specific protocols to enhance DNA extraction yields before PCR assay (56). However, it is well reported that DNA recovering in FFPE specimens may be influenced by several factors, such as formalin quality and concentration, length of fixation, paraffin quality, and temperature (57) leading to nucleic-acid fragmentation (56, 58, 59). As a consequence, DNA in FFPE biopsy is either completely or partially degraded into DNA fragments of 200 bp or less (58). Low HPV viral load in FFPE biopsy samples associated with a large region targeted by the molecular assay used (>200 pb) could be a limiting factor, and in medical practice, this decreased sensitivity could sometimes hamper HPV detection in OPSCC. Since PCR is a very sensitive technique, the risk of a false positive due to contamination is not negligible. It may occur within the specimen by a fragment of normal epithelium infected with an HPV unrelated to the cancer. Contamination may also occur during specimen processing with another sample (cross-contamination) or with a soiled object in the laboratory (60). For all these reasons, HPV diagnosis and genotyping on FFPE biopsy from OPSCC using commercially available HPV molecular assays require a good expertise, particularly for preanalytical treatment. This step could require complementary technical approaches to increase sensitivity, as we recently described (52). For example, since HPV16 is known to be the most prevalent HPV genotype in OPSCC, diagnosed in more than 85% of HPV-driven OPSCC (61), we think that it is better to confirm negative results obtained with certain commercial tests through an HPV16-specific home-made PCR able to detect smaller fragments of DNA (<100 pb) from FFPE samples (52).

#### *In situ* Hybridization Targeting DNA (DNA ISH)

Many studies have evaluated the use of DNA ISH to diagnose HPV infection in oropharynx carcinomas (54, 62–66). This technique is based on the hybridization of probes against specific sequences of DNA, and conventional light microscopy is sufficient to read the assay result. It has the advantage of being cheaper than RNA *in situ* hybridization, but it seems that sensitivities and specificities of this assay strongly depend on the type of probes used to target HPV (e.g., different manufacturers, probe designs). Depending on the DNA targets, DNA ISH can focus only on high-risk 16 and 18 genotypes, or on broader high-risk HPV-like genotypes 16, 18, 31, 33, 51 (Enzo©’s high-risk cocktail here for example; Enzo, NY, United States). Ventana© Inform HPV III Family 16 Probe cocktail is also able to detect 16, 18, 31, 33, 35, 39, 45, 51, 52, 56, 58, and 66 types. Finally, some screening probe cocktails can detect most frequent high-risk HPV (16, 18, 31, 33, 51), as well as some low-risk HPV (6 and 11 types for Enzo©’s screening probe for example).

**FIGURE 1:**
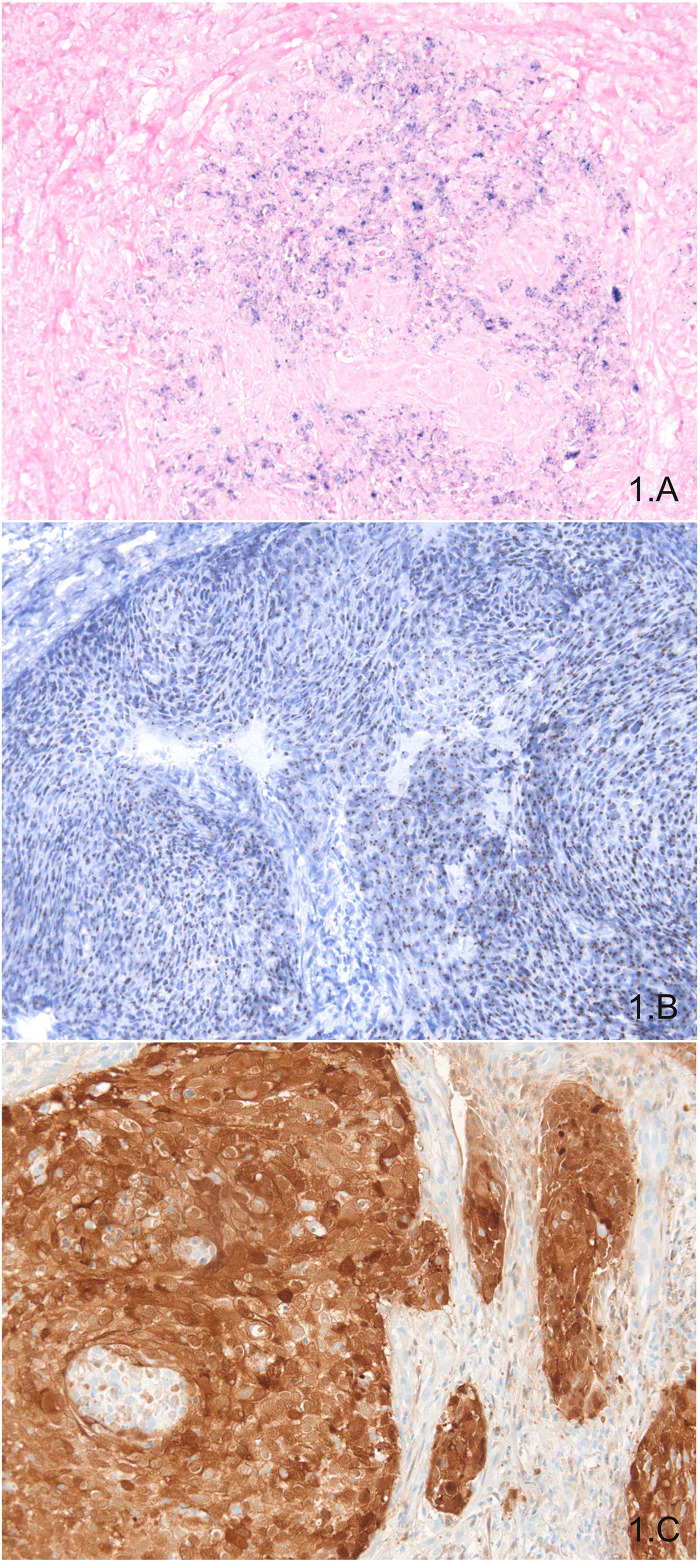
*In situ* exploration of HPV presence: DNA ISH showing blue punctate staining in tumor cells **(A)**; RNA ISH showing brown punctate staining in tumor cells **(B)**; P16 immunohistochemistry showing diffuse and intense nuclear and cytoplasmic staining in almost all tumor cells **(C)**.

Data about consistency of DNA ISH results in OPSCCs are quite controversial. For Schlecht et al., comparison of high-risk HPVs probe cocktail (Ventana©, AZ, United States) and HPV16/18 DNA probe cocktail (Dako©, CA, United States) showed better performances by the first manufacturer (66). Conversely, Keung et al. did not find significant differences between performances of three different manufacturers’ probe: Enzo© (NY, United States), Leica© (Germany), and Ventana© (AZ, United States) (67). It seems that DNA ISH quality is highly dependent on quality control procedures, and experience of the laboratory with this technique should be taken into account (68). Importantly, Bishop et al. reported that an important background signal could hinder the visualization of the punctuate signal corresponding to target DNA and thus lead to false-negative cases (69). More precisely, it seems that when less than 100 copies of target HPV are present in tumor cells, approximately 25–45% of cases would be reported falsely negative (67). For all these technical reasons, the popularity of DNA ISH appears to have come to a standstill whereas RNA ISH interest is surely growing. Figure 1A shows an example of positive DNA ISH targeting HPV in OPSCC.

#### *In situ* Hybridization Targeting RNA (RNA ISH)

Studies about RNA ISH have been rising in the last 10 years and showing excellent diagnostic performances. Sensitivities vary from 87 to 100%, and specificities vary from 88 to 100%, being more frequently around 95% (62–65, 69–72). Importantly, studies using RT-PCR as the reference test found the best diagnostic performances, making RNA ISH the method of choice for detecting high-risk HPV infections (62, 65, 71). The RNAscope© (ACD©, DC, United States) technology is the most used one and gives excellent results. This technology can detect E6 and E7 transcripts from 18 high-risk HPV genotypes (HPV16, 18, 26, 31, 33, 35, 39, 45, 51, 52, 53, 56, 58, 59, 66, 68, 73, and 82). RNA ISH has the advantage of being feasible on paraffin-embedded tissues. In short, RNAscope© has a good specificity thanks to paired “Z” probes system and a good sensitivity thanks to the amplification system. Moreover, the small size of probes used for this assay enables hybridization to partially degraded mRNA, notably in paraffin-embedded tissues (Figure 1B). Another advantage of this method is to be readable on conventional optic light microscope (73). Biologically, RNA ISH is relevant because it addresses several points: (i) the presence of signal indicates the presence of one of the 18 high-risk HPVs included in the probe cocktail, (ii) whether there is a transcriptionally active infection, and (iii) the location of the signal within the tumor cells. Some studies suggest that the analysis of signal could be quantitative or semiquantitative (63, 74, 75), but more studies are necessary to confirm these data. Combining RNA ISH with other assays does not seem to be worthwhile, because it has great diagnostic performances and it would hamper the workflow of specimens using two assays. Nevertheless, to answer that question, a study testing RNA ISH and p16 immunostaining using RT-PCR as the gold standard would be required. In a perspective of clinical routine use of RNA ISH, Kerr et al. compared the diagnostic performances of manual and automated assays in a series of 45 HNSCCs, approximately two thirds being OPSCCs (76). Concordance between manual and automated assays was high (96%). Another study showed the same results with a high concordance between automated and manual RNA ISH, with only 3 cases out of 42 HNSCCs (35 OPSCCs) being discrepant (kappa = 0.915) (77). These data are in favor of the utilization of RNA ISH on automated platforms. This would enhance workflow efficiency in a routine practice with a high volume of specimens.

The main inconvenient of RNA ISH is its cost, rendering this diagnostic option poorly available for numerous pathology laboratories. A secondary limit of this assay is its incapacity to assess which one of the high-risk HPV types is present in the tumor tissue, whereas this information could be useful to precise prognosis of HPV-positive OPSCCs (78).

It has been shown that oropharynx cancers with transcriptionally active HPV infections are genetically different entities and have a better prognosis (79, 80). In practice, it was necessary to ascertain that RNA ISH was able to predict survival of patients, as well as RT-PCR. Studies have shown that *in situ* hybridization is equivalent for appreciation of prognosis compared to RT-PCR. They showed better survival for patients with HPV-driven OPSCC sought by E6/E7 *in situ* hybridization (65, 81–83). Additionally, our team has shown a difference in prognosis within HPV-related OPSCC depending on the intensity of the RNA ISH staining. Over 50 histologically confirmed p16 positive oropharyngeal squamous cell carcinomas, we applied HPV RNA ISH with a E6/E7 high-risk RNA probe. The staining was assessed semiquantitatively to define two scores: RNA ISH “low” and RNA ISH “high.” This series contained 29 RNA ISH low cases (58%) and 21 RNA ISH high cases (42%). RNA ISH high staining was associated with a better overall survival in both univariate and multivariate analyses (*p* = 0.033 and *p* = 0.042, respectively) (84). Nowadays, this technique is not yet recommended to be used routinely and is only applied for research purposes.

#### p16 Immunostaining

Immunostaining against p16 protein is a cost-effective method to diagnose a high-risk HPV infection within tissues. Overexpression of p16 protein may be an indirect sign of expression of E6 and E7 proteins with cell-cycle upregulation (24, 30). Nevertheless, other processes can lead to p16 overexpression: inflammation, regeneration, and p53 mutations (85, 86). Diagnostic performances of p16 immunostaining are considered high enough to diagnose a high-risk HPV infection in oropharyngeal squamous cells carcinomas, and according to the College of American Pathologists and to the eighth edition of the TNM classification, this assay can be used as a surrogate marker of high-risk HPV infection (24). Sensitivity and specificity of p16 immunostaining for high-risk HPV infection vary from approximately 80–98% according to studies. Among other causes, these differences may be explained by the number of cases included for comparison, by the reference test used as gold standard (RT-PCR, PCR, RNA ISH), and by whether tissue microarray (TMA) were used or not. Interestingly, studies using TMA to evaluate diagnostic performances of p16 immunostaining tend to report lower sensitivities (54, 87). This might be explained by intratumoral heterogeneity of p16 immunostaining (88). Chen et al. have shown that a diffuse nuclear and cytoplasmic staining is significantly associated with HPV positivity in OPSCCs regardless of the intensity of staining, contrary to focal nuclear and cytoplasmic staining (89). Nevertheless, this information is difficult to evaluate on biopsies. Concerning subcellular localization, according to Lai et al. and Zhao et al., it seems that OPSCCs associated with a highly intense nuclear and slightly intense cytoplasmic p16 immunostaining have poor prognosis, similarly to p16-negative OPSCCs. In both studies, OPSCCs with a high nuclear and high cytoplasmic p16 immunostaining are confirmed to be significantly associated with a better prognosis (90, 91). Figure 1C shows an example of positive p16 immunohistochemistry with a diffuse and intense nuclear and cytoplasmic staining of most of tumor cells.

Sensitivity of p16 immunostaining in the oropharynx is around 80–90% (8, 62, 63, 70, 71, 81, 82, 87, 88, 92–95). One study compared the performances of p16 immunostaining according to the threshold of positivity used to assess p16 immunostaining positivity (96). The authors show that determining p16 positivity using a 75% threshold is associated with a poor reproducibility, whereas a 50% threshold is more reproducible. Besides, although a 70% threshold is recommended by most institutions (24, 97, 98), several teams have shown that 50–70% of positivity is often consistent with high-risk HPV infection (88). Thus, one could wonder if using a 50% positivity threshold to assess p16 positivity in routine practice might be an effective diagnostic approach. Further studies led in different OPSCC populations and comparing different thresholds of positivity are required. Considering that specificity of p16 immunostaining varies from 80 to 90% (8, 62, 63, 70, 71, 81, 82, 87, 88, 92–95), some patients with OPSCC may be diagnosed as having a transcriptionally active HPV infection when it is not the case. Rietbergen et al. showed especially that OPSCCs with a p16 immunostaining, and no transcripts of E6 and E7 proteins have a poorer prognosis compared to those with E6 and E7 transcripts (86). Using RNA ISH, we have found similar results (63). Using p16 immunostaining alone could misclassify some patients, but in a large scale of OPSCC management, this option makes sense because the assay is affordable and available for many pathologic departments. However, for trials evaluating impact of treatments according to HPV status, this diagnostic option does not seem performant enough for us, and an assay detecting E6 and E7 transcripts could then be used (RT-qPCR if frozen samples are available, RNA ISH if only formalin-fixed paraffin-embedded tissues are available).

Several studies and meta-analyses have shown that p16-positive OPSCCs have a better overall survival and a better disease-free survival compared to p16-negative OPSCCs (5, 99–101), whatever the age of patients (102). Within p16 + OPSCC, it is unclear whether the prognosis is solely related to HPV or whether p16 expression could be a prognostic factor in itself. Indeed, few studies compare the prognosis of p16 + /HPV- OPSCC patients to p16 + /HPV + or p16-/HPV- OPSCC patients: this p16 + /HPV- subgroup in OPSCC most often has a small number of patients, and the results are therefore not representative. The studies are moreover contradictory, demonstrating for some that there is a better prognosis in spite of the expression of p16 alone in OPSCC (100), and for others that there is no difference in prognosis in OPSCC between the p16 + /HPV- and p16-/HPV- subgroups (70, 101, 103). Studies with a higher number of patients are needed to clarify this issue. One caveat about p16 immunostaining is that it does not provide any data about HPV types involved in the oncogenic process, although this information may be important because a recent study suggested that some high-risk HPV types might be associated with a worse prognosis than others. Indeed, Chatfield-Reed et al. showed that compared to HPV16 type, HPV33 type could be independently associated with a shorter survival, making p16 immunostaining suboptimal to predict survival differences within high-risk HPV-positive OPSCCs (78).

As p16 immunostaining is not a good surrogate marker of high risk HPV infection in non-OPSCC (104), it is rational to ask whether this marker is of prognostic interest in these cancers. Studies are contradictory, but those with larger cohorts seem to support an absence of prognostic difference. In over 1362 HNSCC from the United States, Brazil, and Europe, D’souza et al. found that p16-positive cases had a lower risk of death compared to p16-negative cases among non-OP HNSSCs in univariate analysis (HR = 0.74, 95% CI = 0.57–0.96), but it was not confirmed after adjusting for other risk factors (aHR = 0.83, 95% CI = 0.60–1.14) (101). In another cohort of 621 non-OPSCC, Fakhry et al. found a similar result: overall survivals of patients with p16−positive non−OP HNSCC (*n* = 62) and with p16−negative non−OP HNSCC (*n* = 559) were not significantly different (*p* = 0.26) (105). More specifically, regarding laryngeal and hypopharyngeal SCC in a small cohort of 31 patients, there was no significant difference in overall survivals (*p* = 0.34) between the p16-positive and p-16 negative patients (106).

There are few data concerning the response to anti-EGFR treatment according to the p16 status. In locally advanced OPSCC, patients with p16−positive tumors had significant superior OS than those with p16−negative tumors in both cetuximab plus radiotherapy (RT) and RT-alone treatment arms (107). Regarding recurrent or metastatic HNSCC, p16−positive status was associated with better overall survival in both the cetuximab plus platinum plus 5−FU and platinum plus 5−FU treatment arms (108). On the contrary, with the panitumumab in the SPECTRUM study, median overall survival in patients with p16-negative HNSCC was longer in the panitumumab group than in the control group (*p* = 0.0115). This difference was not shown for p16-positive patients (*p* = 0.998) (109).

Finally, concerning the response to immunotherapies there are again few data available, but p16 status is quite consistently used. In KEYNOTE-012, for the head-and-neck cohorts, the percentage of p16 + patients was relatively small with 45 (23%) being p16 + and 147 (77%) being p16- (110). When stratified by p16 status, response rates were higher in p16 + patients compared to p16- patients, with demonstrated ORRs of 24% (95% CI, 13–40%) and 16% (95% CI, 10–23), respectively (110, 111). These results are contradictory with the CheckMate 141 study in which 63 (26%) patients were p16-positive, 50 (21%) were p16-negative, and 127 (53%) were not tested (112). Analyses revealed nivolumab to be beneficial compared to standard-of-care chemotherapy, irrespective of p16 (112). This was confirmed in a recent update, with significant benefit in both p16- patients and p16 + patients (113).

## New HPV Biomarkers in the Management of HPV-Driven OPSCC

Completing these recommended routine diagnostic tests used to properly classify OPSCC due or not to HPV infection, other new performant biomarkers seem to be useful for HPV-induced OPSCC ultrastaging. Indeed, as we already described before, the E6 and E7 HPV oncoproteins are involved in cell transformation and carcinogenesis and have been proven to be indispensable for maintenance of tumor phenotype (32). Moreover, recent *in vitro* data suggest that E6 and E7 oncoproteins and spliced isoforms of E6 oncoprotein would be associated with higher levels of IL6, responsible of an immunosuppressive environment within cancer (114). This immunosuppressive context could be targeted by therapies associating IL6 and PD-1/PD-L1 blockade (115). To our knowledge, HPV-derived nucleic acids, and particularly the E6 and E7 genes, have not been detected in blood samples in case of simple HPV mucosal infection but only in HPV-related cancer cases (116). Therefore, HPV circulating tumoral DNA (ctDNA) based on detection of HPV DNA in plasma with new ultrasensitive methods appears to have a clinical interest in HPV OPSCC (52, 117). The detection of humoral response against HPV early proteins, especially antibodies against E6, has also been associated with an increased risk to develop oropharyngeal cancer (118).

### E6 Humoral Response

The detection of humoral response against HPV early proteins, particularly antibodies against E6 protein, has been associated with a 132-fold increase risk to develop oropharyngeal cancer (118). Rather interestingly, these antibodies seem to develop more than 10 years before HPV-driven OPSCC diagnosis (119). Meanwhile, these E6 antibodies are detectable in <1% of healthy controls (120, 121). Finally, different studies have shown that the vast majority of HPV-positive OPSCC patients (>90%) present an HPV16 E6 antibody response in blood at the time of their HPV16-OPSCC diagnosis (119–124). Even if some authors argue that E6 serology could be helpful for HPV OPSCC monitoring, particularly to track residual disease or recurrence (125), its interest must be confirmed and validated before considering its general use in clinical routine. Even if baseline HPV16 E6 antibodies may have a potential clinical utility for the diagnosis and/or prognosis of HPV-induced OPSCC because HPV16 E6 seropositivity is associated with significant reduced risk of recurrence, E6 serology does not represent a good biomarker for posttreatment monitoring and early identification of relapses. Indeed, HPV16 E6 antibody level remains stable in patients after treatment and eventual variations in antibodies level were not associated with recurrence (126).

### HPVct DNA by ddPCR

As we previously mentioned, HPV circulating tumoral DNA (ctDNA) based on detection of plasmatic HPV DNA (E6 or E7 genes) with new ultrasensitive methods appears to have a clinical interest in HPV OPSCC. Indeed, the liquid-biopsy approach using the detection of ctDNA released from tumor cells and detectable in blood has garnered growing interest (127) particularly in HNSCC (128). Detection of ctDNA has demonstrated its relevance in lung or colorectal cancer with the detection of *EGFR* and *KRAS* mutations for non-invasive tumors genotyping, treatment response follow-up, and relapse prediction (129). HPV-related cancers are an ideal model to monitor ctDNA by detecting HPV oncogenes E6 or E7. The feasibility and the interest of HPV ctDNA detection in the plasma of HPV-related OPSCC patients using new ultrasensitive molecular tools such as droplet-based digital PCR (ddPCR) assays have been recently reported and correlated with clinical outcome (52, 117) and early detection of recurrences in posttreatment monitoring (130). This quantitative method of ddPCR is characterized by its high sensitivity, its accuracy, and its reproducibility inter- and intra-laboratories (131). Our team has recently highlighted the interest of quantifying HPVctDNA in plasma samples of OPSCC patients at baseline (52). Indeed, it is the first time that pre-therapeutic HPVctDNA using ddPCR technology was evaluated as a biomarker for OPSCC staging correlated with the new AJCC staging algorithm for HR HPV-associated OPSCC (132) and for patients’ clinical outcome. We demonstrated a positive correlation between the level of HPVctDNA load quantified by ddPCR and T status, N status, and the specific stages of the new HPV OPSCC staging algorithm. Moreover, in our series, we observed a positive correlation between HPVctDNA detection by ddPCR and patient clinical outcome. Even if further studies need to be performed in larger cohorts to confirm the prognostic interest of this biomarker before considering its use in routine practice, HPVctDNA appears to be a very interesting biomarker to monitor for optimization of HPV-related OPSCC management with potential interest to select patients for whom treatment de-escalation could eventually be offered.

Finally, the performance of HPVctDNA has also been evaluated to monitor treatment response early, showing that HPVctDNA kinetics are clearly correlated with treatment failure or success and this feature would be more precocious than classical Response Evaluation Criteria in Solid Sumours (RECIST) criteria (52, 117, 133). In the future, the monitoring of HPVctDNA could also be considered as an easy-to-use plasmatic biomarker to determine treatment efficacy early considering the increasing use of very specific and expensive treatment such as immunotherapies in OPSCC medical support. According to the different studies already published on HPVctDNA in HPV-driven OPSCC, this biomarker has a very high sensitivity and specificity, recently estimated at 89 and 97%, respectively by Chera et al. (133). Finally, another great interest of the quantification of HPVctDNA by ddPCR is its very low cost compared to other innovative technologies.

### HPV Capture Technology and Viral Molecular Signatures

In cervical carcinomas, integration of HPV DNA into the host genome seems to be the main critical etiological event in the progression from normal cervix to intraepithelial neoplasm, and finally to invasive cervical cancer. This HPV oncogenic process is considered to be identical in OPSCC, but with no scientific certainties as pretumoral lesions are not yet characterized in head and neck cancer. However, for cervical cancers, different studies have already shown that a part of HPV-driven tumor does not present any integration and is associated only with episomal HPV (134–136). Therefore, HPV molecular status (integrated or not) in the tumor cells could represent an interesting profile to clarify and to correlate with clinical data. Moreover, if integration occurred, the site of HPV integration could also have a real impact on cancer progression (disruption of cancer suppressor genes, immunomodulatory genes, etc.). Finally, HPV genotype variant description could also be of interest as HPV variants have been shown to differ biologically and functionally, thereby affecting persistence and potentially the risk of progression (137, 138). Identifying HPV genotype variants could be pertinent to classify them according to their tumoral aggressiveness.

Recently, using a next-generation sequencing (NGS) technology called “Capture HPV” (135) on biopsies and circulating DNA material, five molecular signatures of HPV integration have been identified in HPV cervical cancer and correlated with survival (but not significantly). To describe the molecular HPV profile and variants in tumor samples, this new and innovative “HPV capture” technology is based on a generic and comprehensive HPV genome capture (235 genotypes and variants) followed by NGS. Exhaustive data will be obtained as HPV whole-genome sequencing/HPV molecular status (integrated or episomal)/HPV integration site, both in virus and human genomes/HPV genotype variant sequences.

“HPV capture” technology has already been performed on HPV cervical and anal cancers (135, 139) to determine a potential prognosis value of the HPV molecular signatures described. Investigations based on this new technology are actually in process in HPV oropharyngeal cancer. The deep information obtained with such technology such as viral molecular status, genotype variants, integration of viral genes deletion, and sites of integration could be extremely informative regarding the viral oncogenic process and could allow the possibility to ultrastratify HPV-driven OPSCC based on virological information.

### Which Sample for Which Test?

Depending on the material obtained from patients, different HPV assays are feasible. Some samples require more invasive procedures than others. For this reason, except for the specific context of a clinical trial, performing a second “fresh” biopsy for RT-PCR is not standard because it requires invasive procedures. The new generation of HPV assays is highly sensitive and can be performed on non-invasive or minimally invasive samples, such as blood puncture and oral rinse. These approaches will undoubtedly be complementary to current classical routine practice HPV assays and will help to stratify and monitor HPV-positive HNSCCs. Considering the availability of human samples and technical aspects of assays cited above, we have briefly, through this review, given an overview of the techniques feasible on each kind of sample.

## Conclusion

In this review, we have explored main HPV detection tools available in routine practice on fresh, frozen, and formalin-fixed tissues in the HNSCC context. If p16 immunostaining is the most affordable technique, it seems that the threshold of 70% of positive tumor cells recommended by the College of American Pathologists might be a little too high because a fraction of cases with a nuclear and cytoplasmic staining in 50–70% of tumor cells are clearly associated with high-risk HPV infection. Of the two *in situ* hybridization assays, the popularity of RNA ISH stems from its excellent diagnostic performances and the biological value of the assay, because positive cases show evidence of transcriptionally active HPV infection. Nevertheless, the price of this assay hampers its use in routine practice. DNA ISH is more difficult to read, and the technique process is highly dependent on the level of expertise of pathology laboratories. This variability leads to moderate diagnostic performances, and this assay is becoming unpopular. RT-PCR and PCR are non-spatial assays but are powerful tools to detect HPV infection. RT-PCR is more performant on fresh and frozen tissues which are often not available in routine practice. For PCR, several commercial assays have been developed for cervical cancers and could be used for HNSCCs, but an important work of comparative evaluation of these tools is needed in HNSCCs and some pre-PCR steps might be optimized to enhance the yield of the technique. Pragmatically, the high sensitivity of p16 immunostaining and the value of PCR to specify HPV type make these tools really interesting in routine practice. Indeed, using p16 immunostaining as a screening tool than PCR constitutes a performant way to diagnose and specify the HPV type since this information is important because of its prognostic value even among high-risk HPV types. In case RNA ISH is feasible, using it as a standalone test might be a seductive solution but it does not provide any precision on the HPV type. We think that further studies evaluating the impact of high-risk HPV type in the prognosis of patients should be conducted to be sure that this information requires a second PCR assay. Among new HPV biomarkers, HPVctDNA detection could be a useful monitoring tool to detect early disease recurrence. This latter tool also seems to have prognostic value, since quantification of HPVctDNA is correlated with T and N stages in OPSCCs. Finally, HPV capture, based on next-generation sequencing, gives insights into the integration process of various genotypes of HPV. In the near future, this assay could be a stratification and prognostic tool for patients with HPV-induced OPSCC.

## Author Contributions

JA, CL, and CBa designed the manuscript. JA, CL, and HP wrote the first draft. AM and AB illustrated the manuscript. JA, CL, CBa, AM, AB, DV, CBr, HP, and HM edited and revised the manuscript. All authors contributed to the article and approved the submitted version.

## Conflict of Interest

CBa is consultant for Roche, MSD and HP is consultant for MSD Vaccin. The remaining authors declare that the research was conducted in the absence of any commercial or financial relationships that could be construed as a potential conflict of interest.
